# Metabolic engineering of *Corynebacterium glutamicum* for efficient production of succinate from lignocellulosic hydrolysate

**DOI:** 10.1186/s13068-018-1094-z

**Published:** 2018-04-04

**Authors:** Yufeng Mao, Guiying Li, Zhishuai Chang, Ran Tao, Zhenzhen Cui, Zhiwen Wang, Ya-jie Tang, Tao Chen, Xueming Zhao

**Affiliations:** 1 Key Laboratory of Systems Bioengineering (Ministry of Education), SynBio Research Platform, Collaborative Innovation Center of Chemical Science and Engineering (Tianjin), School of Chemical Engineering and Technology, Tianjin University, Tianjin, 300072 China; 2 Key Laboratory of Fermentation Engineering, Ministry of Education, Hubei University of Technology, Wuhan, 430068 China

**Keywords:** *Corynebacterium glutamicum*, Succinate, Xylose, Lignocellulosic hydrolysate

## Abstract

**Background:**

Succinate has been recognized as one of the most important bio-based building block chemicals due to its numerous potential applications. However, efficient methods for the production of succinate from lignocellulosic feedstock were rarely reported. Nevertheless, *Corynebacterium glutamicum* was engineered to efficiently produce succinate from glucose in our previous study.

**Results:**

In this work, *C. glutamicum* was engineered for efficient succinate production from lignocellulosic hydrolysate. First, xylose utilization of *C. glutamicum* was optimized by heterologous expression of *xylA* and *xylB* genes from different sources. Next, *xylA* and *xylB* from *Xanthomonas campestris* were selected among four candidates to accelerate xylose consumption and cell growth. Subsequently, the optimal *xylA* and *xylB* were co-expressed in *C. glutamicum* strain SAZ3 (Δ*ldhA*Δ*pta*Δ*pqo*Δ*cat*P_sod_-*ppc*P_sod_-*pyc*) along with genes encoding pyruvate carboxylase, citrate synthase, and a succinate exporter to achieve succinate production from xylose in a two-stage fermentation process. Xylose utilization and succinate production were further improved by overexpressing the endogenous *tkt* and *tal* genes and introducing *araE* from *Bacillus subtilis.* The final strain *C. glutamicum* CGS5 showed an excellent ability to produce succinate in two-stage fermentations by co-utilizing a glucose–xylose mixture under anaerobic conditions. A succinate titer of 98.6 g L^−1^ was produced from corn stalk hydrolysate with a yield of 0.87 g/g total substrates and a productivity of 4.29 g L^−1^ h^−1^ during the anaerobic stage.

**Conclusion:**

This work introduces an efficient process for the bioconversion of biomass into succinate using a thoroughly engineered strain of *C. glutamicum*. To the best of our knowledge, this is the highest titer of succinate produced from non-food lignocellulosic feedstock, which highlights that the biosafety level 1 microorganism *C. glutamicum* is a promising platform for the envisioned lignocellulosic biorefinery.

**Electronic supplementary material:**

The online version of this article (10.1186/s13068-018-1094-z) contains supplementary material, which is available to authorized users.

## Background

In 2004, the US Department of Energy (DOE) designated succinate as one of the 12 most promising bio-based platform chemicals [1]. Succinate and its derivatives have extensive applications in the chemical, food, agricultural, and plastics industries [2]. The demand for this valuable building block is expected to rapidly increase to 700 000 tons per year by 2020 [3]. As reviewed by Ahn et al. [4], great advances have been made in the development of metabolically engineered microorganisms capable of efficiently producing succinate on an industrial scale. *Corynebacterium glutamicum* S071/pGEX4-NCgl0275 was even able to produce 152.2 g L^−1^ succinate from glucose [5], which is the highest succinate titer ever reported. Moreover, wild-type *Actinobacillus succinogenes* ATCC55618 was reported to be able to produce 151.44 g L^−1^ succinate from fresh cassava root [6]. However, studies of succinate production over the last decade have focused on the use of purified sugars or food-based feedstocks, which cannot be used industrially. There also seem to be few reports on the efficient production of succinate from non-food lignocellulosic feedstocks, such as corn stalk, sugarcane bagasse, pine-, oak-, or spruce wood. Nevertheless, some representative studies with good performance do exist. For example, 11.13 g L^−1^ of succinate was produced from corn stalk hydrolysate with a high yield of 1.02 g/g total sugars using *E. coli* BA204 [7]. The strain *E. coli* SD121 was able to produce 57.8 g L^−1^ succinate from corn stalk hydrolysate with a yield of 0.87 g/g total sugars [8]. Moreover, *A. succinogenes* NJ113 was able to produce 70.3 g L^−1^ of succinate from corn fiber hydrolysate with a yield of 0.68 g g^−1^ total sugars [9]. Up to now, the highest succinate titer from non-food lignocellulosic hydrolysate was produced from sugarcane bagasse hydrolysate using *E. coli* BA305, which reached 83 g L^−1^ by summing up the titers of three repetitive fermentations [10]. However, the process of cell recycling is complicated and costly. In most reports on succinate production from lignocellulosic biomass, the yields were quite good, but the titers were generally limited by the sugar concentration obtained from the hydrolysis of lignocellulosic biomass, making the process uneconomical for further separation and purification. Hydrolysis efficiency is, therefore, considered a key factor of process viability. Enzymatic hydrolysis combined with dilute acid pretreatment was utilized as the primary method for sugar production from lignocellulosic biomass. Corn stalk is an agricultural byproduct of annual renewable crops and is available in abundance around the world. In fact, it is estimated that approximately 230 million tons of lignocellulosic corn stalks are produced each year [11]. Corn stalk biomass is, therefore, a potential feedstock for biorefineries in China. In this study, corn stalk hydrolysates containing high concentrations of glucose and xylose obtained by means of enzymatic hydrolysis were chosen as substrate for succinate production.

*Corynebacterium glutamicum*, a facultatively anaerobic Gram-positive soil bacterium with GRAS (generally regarded as safe) status, is one of the major succinate producing organisms [2, 12]. Under oxygen deprivation, energy and carbon will primarily be channeled towards product formation rather than growth in *C. glutamicum* [13]. A series of metabolically engineered *C. glutamicum* strains were constructed for the efficient production of succinate from glucose under anaerobic conditions [5, 13–17]. The succinate production processes that utilize *C. glutamicum* were mainly based on glucose from starch. Strain NC-2, with an *ldhA* deletion and the introduction of *xylA* and *xylB* genes from *E. coli*, produced 40.8 g/L of succinate in 48 h from corn cob hydrolysate with a yield of 0.69 g g^−1^ total sugar under anaerobic conditions [18]. However, the titer and yield were relatively low and still not satisfactory.

Xylose utilization is considered a cornerstone of the efficient utilization of lignocellulosic biomass. Because of the lack of a xylose isomerase (XI) pathway, *C. glutamicum* could not use xylose as the carbon source before xylose isomerase from *E. coli* was introduced [19]. Since then, a variety of metabolic engineering strategies have been studied and applied to improve xylose utilization. These include multiple chromosomally integrated copies of the *xylAB* operon [20], expression optimization of assimilation pathways [21, 22], selection of xylose isomerase and xylulokinase [23], introduction of a heterogenous xylose transporter [24–27], and overexpression of a non-oxidative pentose phosphate pathway (PPP) to increase the flux through the xylose metabolism [28]. Radek et al. [29] engineered a *C. glutamicum* strain showing more carbon efficiency but a lower growth rate, by converting xylose into α-ketoglutarate via the Weimberg pathway, which was beneficial for succinate yields under aerobic conditions [30]. However, the XI pathway was still the main pathway used in succinate production from xylose under anaerobic conditions.

**Fig. 1:**
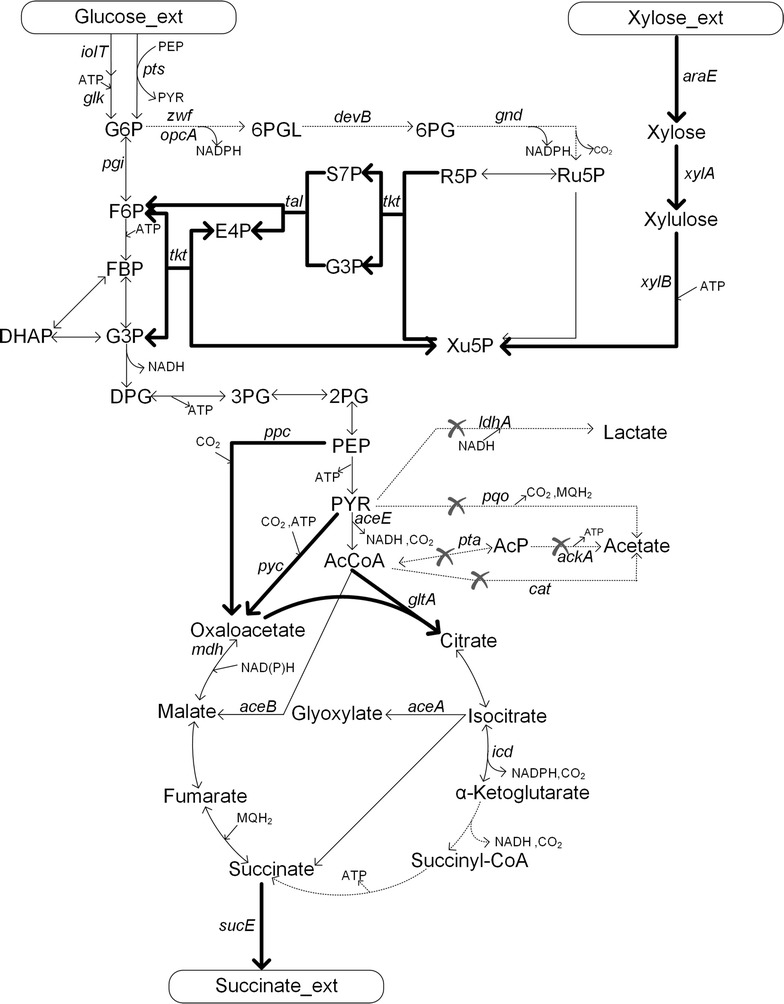
Succinate biosynthesis pathway of *C. glutamicum*. The bold black arrows indicate metabolic fluxes increased by overexpression or introduction of the corresponding genes. The gray arrows indicate the reactions leading to a byproduct or presumably irrelevant reactions. Deleted genes are indicated with crosses. Metabolites: G6P glucose-6-phosphate, 6PGL 6-phosphoglucono-1,5-lactone, 6PG 6-phosphogluconate, Ru5P ribulose-5-phosphate, Xu5P xylulose-5-phosphate, R5P ribose-5-phosphate, G3P glyceraldehyde-3-phosphate, S7P sedoheptulose-7-phosphate, F6P fructose-6-phosphate, FBP fructose-1,6-bisphosphate, E4P erythrose-4-phosphate, DHAP dihydroxyacetone, DPG glycerate-1,3-diphosphate, 3PG glycerate-3-phosphate, 2PG glycerate-2-phosphate, PEP phosphoenolpyruvate, PYR pyruvate, AcP acetyl phosphate, AcCoA acetyl-CoA. Genes and their encoded enzymes: *iolT* encoding myo-inositol permease, *glk* encoding glucokinase, *ptsG* encoding glucose-EII of phosphoenolpyruvate phosphotransferase system (PTS), *pgi* encoding glucose-6-phosphate isomerase, *araE* encoding a H^+^ symporter protein, *xylA* encoding xylose isomerase, *xylB* encoding xylulokinase, *zwf* and *opcA* encoding glucose-6-phosphate dehydrogenase, *devB* encoding 6-phosphogluconolactonase, *gnd* encoding 6-phosphogluconate dehydrogenase, *tkt* encoding transketolase, *tal* encoding transaldolase, *pqo* encoding pyruvate: quinone oxidoreductase, *pta* encoding phosphotransacetylase, *ackA* encoding acetate kinase, *cat* encoding acetyl-CoA:CoA transferase, *aceE* encoding pyruvate complex dehydrogenase E1 component, *ppc* encoding phosphoenolpyruvate carboxylase, *pyc* encoding pyruvate carboxylase, *mdh* encoding malate dehydrogenase, *gltA* encoding citrate synthase, *sucE* encoding succinate exporter

In the present study, several strategies were taken into account to improve xylose utilization in *C. glutamicum.* The engineered *C. glutamicum* SAZ3 was selected as the chassis for succinate production. The final engineered strain CGS5 (Fig. 1) could simultaneously and completely consume sugar mixtures comprising glucose and xylose under anaerobic conditions. Using enzymatic hydrolysate of corn straw, it produced 98.6 g L^−1^ succinate with a yield of 0.87 g g^−1^ total substrates in the anaerobic stage. These are the highest titers obtained with non-food lignocellulosic hydrolysate as the substrate to date.

## Methods

### Strains and media

All the strains used in this study are listed in Table 1. *Escherichia coli* DH5α (Invitrogen) was used for routine cloning and maintenance of plasmids. The succinate-producing *C. glutamicum* strain SAZ3 [31], with deletions of *ldhA*, *pqo*, *cat,* and *pta*, and replacement of the native promoters of *pyc* and *ppc* with the *sod* promoter (Δ*ldhA*Δ*pta*Δ*pqo*Δ*cat*P_sod_-*ppc*P_sod_-*pyc*), was used as host strain.

**Table 1:** Strains and plasmids used in this study

Strain/plasmid	Relevant characteristics	References
ATCC 13032	*C. glutamicum* wild-type biotin auxotrophic	ATCC
I-pXMJ19	ATCC13032 (pXMJ19)	This study
I-eco	ATCC13032 (pX-ecoAB)	This study
I-sco	ATCC13032 (pX-scoAB)	This study
I-ppm	ATCC13032 (pX-ppmAB)	This study
I-xcb	ATCC13032 (pX-xcbAB)	This study
SAZ3	ATCC13032 ∆*ldh*∆*ackA*-*pta*∆*pqo*∆*cat P*_*sod*_ *pyc P*_*sod*_ *ppc*	Lab stock
CGS1	SAZ3; pX-xcbAB, pEpycgltAsucE	This study
CGS2	ATCC13032 ∆*ldh*∆*ackA*-*pta*∆*pqo*∆*cat P*_*sod*_ *pyc P*_*sod*_ *ppc P*_*sod*_ *tal P*_*sod*_ *tkt*	This study
CGS3	CGS2; pX-xcbAB, pEpycgltAsucE	This study
CGS4	ATCC13032 ∆*ldh*∆*ackA*-*pta*∆*pqo*∆*cat P*_*sod*_ *pyc P*_*sod*_ *ppc P*_*sod*_ *tal P*_*sod*_ *tkt P*_*tuf*_ *araE*	This study
CGS5	CGS4; pX-xcbAB, pEpycgltAsucE	This study
Plasmids		
pDsacB	Kan^R^; vector for in-frame deletion (*sacB*_*B.sub*_; *lacZα*; *OriV*_*E.c*_)	Lab stock
pD-ldh	pDsacB carrying the flanking sequences of the *ldh* gene	This study
pD-PSL	pDsacB carrying the *sod* promoter, upstream and initial sequences of the *tal* gene	This study
pD-PSK	pDsacB carrying the *sod* promoter, upstream and initial sequences of the *tkt* gene	This study
pD-P_tuf_araE	pDsacB carrying *araE* from *B. subtilis* 168 under the control of *P*_*tuf*_, the flanking sequences of the *ldh* gene	This study
pXMJ19	Cm^R^, *C. glutamicum*/*E. coli* shuttle vector (*P*_*tac*_, *lacI*^q^, pBL1, *rrnB*T2T1 terminator, *OriV*_*C.g*_, *OriV*_*E.c*_)	Lab stock
pX-ecoAB	Derived from pXMJ19, for the overexpression of *xylAB* from *E. coli* MG1655	This study
pX-scoAB	Derived from pXMJ19, for the overexpression of *xylAB* from *Streptomyces coelicolor*	This study
pX-ppmAB	Derived from pXMJ19, for the overexpression of *xylAB* from *Paenibacillus polymyxa*	This study
pX-xcbAB	Derived from pXMJ19, for the overexpression of *xylAB* from *Xanthomonas campestris pv. campestris* 8004	This study
pEC-XK99E	Kan^R^; *C. glutamicum*/*E. coli* shuttle vector (*P*_*trc*_, *lacIq*; pGA1, *OriV*_*C.g*_., *OriV*_*E.c*_)	Lab stock
pEpycgltAsucE	Derived from pEC-XK99E, for the overexpression of *pyc*, *gltA* and *sucE*	Lab stock
pEP2	Kan^R^; *C. glutamicum*/*E. coli* shuttle vector, low copy	Lab stock
pEP-P_tuf_araE	Derived from pEP2, for the overexpression of *araE* from *B. subtilis* 168 under the control of *P*_*tuf*_	This study

*ATCC* American type culture collection

Brain heart infusion sorbitol (BHIS) medium was used for transformation of *C. glutamicum*. Lysogeny broth (LB) containing tryptone 10 g L^−1^, yeast extract 5 g L^−1^, and NaCl 10 g L^−1^ was used for cultivation of *E. coli* DH5α. The modified CGIII complex medium (pH 7.4) containing tryptone 10 g L^−1^, yeast extract 10 g L^−1^, and 3-morpholinopropanesulfonic acid (MOPS) 21 g L^−1^ with 20 g L^−1^ glucose was used for precultures of *C. glutamicum* [31]. CGXIIA medium (pH 7.0), containing (NH_4_)_2_SO_4_ 20 g L^−1^, urea 5 g L^−1^, KH_2_PO_4_ 1 g L^−1^, K_2_HPO_4_ 1 g L^−1^, MgSO_4_·7H_2_O 0.25 g L^−1^, CaCl_2_ 10 mg L^−1^, FeSO_4_·7H_2_O 10 mg L^−1^, MnSO_4_·H_2_O 0.1 mg L^−1^, ZnSO_4_·7H_2_O 1 mg L^−1^, CuSO_4_·5H_2_O 0.2 mg L^−1^, NiCl_2_·6H_2_O 20 μg L^−1^, biotin (Sangon Biotech, Shanghai, China) 0.2 mg L^−1^, and MOPS 21 g L^−1^ with the indicated amounts of glucose (named CGXIIA-G) or xylose (named CGXIIA-X) was used during the aerobic stage. CGXIIB medium (pH 7.0), containing (NH_4_)_2_SO_4_ 5 g L^−1^, urea 5 g L^−1^, KH_2_PO_4_ 1 g L^−1^, K_2_HPO_4_ 1 g L^−1^, MgSO_4_·7H_2_O 0.25 g L^−1^, CaCl_2_ 10 mg L^−1^, FeSO_4_·7H_2_O 10 mg L^−1^, MnSO_4_·H_2_O 0.1 mg L^−1^, ZnSO_4_·7 H_2_O 1 mg L^−1^, CuSO_4_·5 H_2_O 0.2 mg L^−1^, NiCl_2_·6 H_2_O 20 μg L^−1^, and biotin 0.2 mg L^−1^ with the indicated amounts of glucose and/or xylose was used as medium for succinate production during the anaerobic stage. Antibiotics were added to the media at the following concentrations when required: 50 μg kanamycin mL^−1^ and 6 μg chloramphenicol mL^−1^ for *E. coli*, 25 μg kanamycin mL^−1^ and 10 μg chloramphenicol mL^−1^ for *C. glutamicum*. Antibiotics and other chemicals used in this study were purchased from Sangon Biotech (Sangon Biotech, Shanghai, China). Corn stalk hydrolysate was obtained from HEBABIZ Pharmaceutical Co. Ltd., China. Before using as substrate for succinate production, the hydrolysate was sterilized at 115 °C for 10 min.

### Construction of plasmids and strains

All the strains and plasmids used in this study are listed in Table 1. All the primers used in this study are listed in Additional file 1: Table S1.

For the heterologous expression of genes encoding xylose isomerase (*xylA*) and xylulokinase (*xylB*) from *Escherichia coli* MG1655, *Paenibacillus polymyxa* SC2, *Streptomyces coelicolor,* and *Xanthomonas campestris* 8004, the plasmids pX-ecoAB, pX-ppmAB, pX-scoAB, and pX-xcbAB were constructed, respectively.

For the construction of plasmid pX-ecoAB, the *xylA* and *xylB* genes were amplified from genomic DNA of *E. coli* MG1655 by PCR using the primer pair ecoxylAB-1/ecoxylAB-2. The resulting fragment was digested with *Hin*dIII and *Xba*I, and ligated into pXMJ19 cut with the same enzymes. The plasmid pX-ppmAB was constructed analogously, using *Xba*I and *Kpn*I.

For the construction of plasmid pX-xcbAB, the *xylA* and *xylB* genes were amplified from genomic DNA of *X. campestris* by PCR using the primer pairs xcbxylA-1/xcbxylA-2 and xcbxylB-1/xcbxylB-2, respectively. The two PCR products were spliced using the primer pair xcbxylA1/xcbxylB2, and the resulting fused PCR product was digested with *Hin*dIII and *Xba*I, and ligated into pXMJ19 cut with the same enzymes. The plasmid pX-scoAB was constructed analogously, using *Pst*I *and Kpn*I.

The replacement of the native promoter of *tal* by the strong *sod* promoter in the recombinant strain SAZ3 was achieved via a two-step homologous recombination procedure using the suicide vector pDsacB. The flanking sequences of the *tal* gene were amplified from genomic DNA of *C. glutamicum* using primer pairs PSL-1/PSL-2 and PSL-3/PSL-4. The *sod* promoter region (192 bp upstream of the *sod* gene) was amplified from chromosomal DNA of *C. glutamicum* using the primer pair P_sod_-1/P_sod_-2. The plasmid pD-PSL was assembled by circular polymerase extension cloning (CPEC) [32], using the overlap of the flanking sequences of the *tal* gene, P_sod_ and pDsacB cut with *Xba*I. The resulting plasmid pD-PSL was used to transform SAZ3 by electroporation. The procedure of promoter replacement was carried out as described previously [14]. Subsequently, the native promoter of *tkt* was analogously replaced by the strong *sod* promoter.

For the construction of the integrating plasmid pD-ldh, the fragment ldhF upstream of the *ldh* gene was first amplified from genomic DNA of *C. glutamicum* 13032 by PCR using the primer pair ldhF1/ldhF2. The PCR product was digested with *EcoR*I and *Sal*I and ligated into pDsacB cut with the same enzymes. Similarly, the fragment *ldhB* downstream of the *ldh* gene was ligated into the plasmid containing *ldhF* cut with *Sal*I/*Hin*dIII.

For the construction of plasmid pEP-P_tuf_araE, the *tuf* promoter region (200 bp upstream of the *tuf* gene) was first amplified from chromosomal DNA of wild-type *C. glutamicum* using the primer pair P_tuf_-1/P_tuf_-2. The PCR product was digested with *Eco*RI and *Sal*I and ligated into pEP2 cut with the same enzymes. Then, the 1.7-kb DNA fragment of the *araE* gene was amplified from chromosomal DNA of *B. subtilis* 168 by PCR using the primer pair araE-1/araE-2. The PCR product was digested with *Xba*I and *Sal*I and ligated into pEP-P_tuf_ cut with the same enzymes.

Chromosomal integration of the xylose transporter gene *araE* was achieved using the plasmid pD-P_tuf_araE. The DNA fragment P_tuf_-araE was amplified from pEP-P_tuf_araE, in which the *araE* gene is under the control of the constitutive *tuf* promoter. The resulting plasmid pD-P_tuf_araE was integrated into the genome of CGS2 by electroporation.

### Culture conditions

For the precultures of *C. glutamicum*, single colonies were grown in 5 mL of BHIS medium [33] at 30 °C and 220 rpm overnight, after which the entire resulting culture was used to inoculate 50 mL of modified CGIII medium supplemented with 10 g L^−1^ of glucose, and grown to an OD_600_ of 10.

For aerobic growth, cells were washed twice with CGXIIB medium and used to inoculate 50 ml of CGXIIA medium with 20 g L^−1^ of xylose or glucose to an initial OD_600_ = 0.8, and cultured in 500 mL shake flasks at 30 °C and 220 rpm on a rotary shaker.

A two-stage process was performed for succinate production. When the cultures reached an OD_600_ of 20, the cells were harvested by centrifugation (5 000×*g*, 4 °C, 10 min), washed with CGXIIB medium. An appropriate amount of washed cells was suspended in 25 mL CGXIIB medium with the indicated sugars and 200 mM sodium bicarbonate, and the cell suspension was cultured in 50 mL serum bottles at 30 °C and 220 rpm on a rotary shaker. To prevent acidification, 30 g L^−1^ of magnesium carbonate hydroxide (4MgCO_3_·Mg(OH)_2_·5H_2_O) was added as a buffering agent. For induction, isopropyl β-d-1-thiogalactopyranoside (IPTG; Sangon Biotech, China) was added to a final concentration of 1 mM. All the fermentation experiments were performed in triplicate.

### Analytical techniques

Extracellular organic acids and pentoses were measured by HPLC [34]. Glucose was monitored using an SBA sensor machine (Institute of Microbiology, Shandong, China). Growth was determined by measuring the optical density at 600 nm (OD_600_). SDS-polyacrylamide gel electrophoretic (PAGE) analysis of intracellular proteins was conducted as previously described [35]. The specific growth rate was calculated as: (ln*W*2 − ln*W*1)/(*t*2 − *t*1), where *W*1 and *W*2 are biomass at times *t*1 and *t*2. The sugar consumption rate was calculated as: *C*_*t*1_/*t*1, where *C*_*t*1_ is the concentration of total consumed sugar at time *t*1. Codon usage analysis was based on the Graphical codon usage analyzer v. 2.0 (http://gcua.schoedl.de/index.html).

### Real-time quantitative PCR (RT-qPCR)

RT-qPCR was conducted as described previously [15]. After 3 h of anaerobic fermentation, the cells were harvested for RNA isolation. The 16S rRNA gene was used as an internal reference for normalization. The primers used for RT-qPCR are listed in Additional file 1: Table S1. Samples were analyzed as previously described [31].

## Results and discussion

### Selection of optimal xylose isomerases and xylulokinases for heterologous expression

**Fig. 2:**
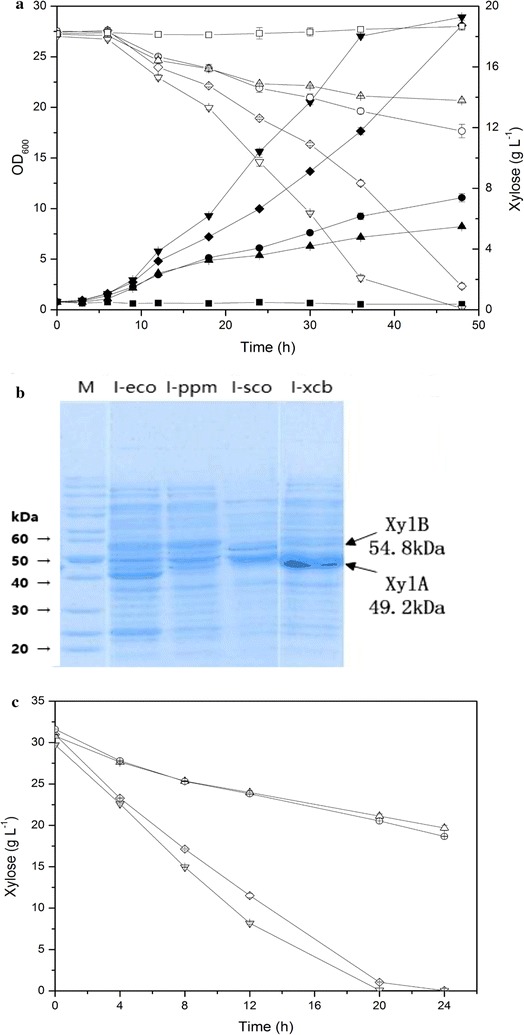
Batch cultivation of *C. glutamicum* I-pXMJ19 (squares), I-eco (diamonds), I-ppm (circles), I-sco (upward triangles), and I-xcb (downward triangles). **a** Profiles of cell growth (filled symbols) and xylose consumption (open symbols) under aerobic conditions. The strains were cultured in CGXIIA medium containing 20 g L^−1^ xylose in 500-mL flasks at 30 °C and 220 rpm with an initial OD_600_ of 0.8, and were induced with 0.5 mM IPTG. **b** SDS–PAGE analysis of intracellular proteins extracted from I-eco, I-ppm, I-sco and I-xcb from cultures with an OD_600_ of 5. Proteins were separated on a 12% SDS-PAGE gel. **c** Profiles of xylose consumption (open symbols) under anaerobic conditions with an initial OD_600_ of 30. 30 g L^−1^ 4MgCO_3_·Mg(OH)_2_·5H_2_O, 200 mM sodium bicarbonate and 1 mM IPTG were added into the CGXIIB medium

Heterologous expression of the XI pathway from *E. coli* endowed *C. glutamicum* with the ability to grow on xylose [19, 23]. However, its xylose utilization capabilities were still unsatisfactory. Hence, selection of a better candidate pathway was carried out in this study. The *xylA* and *xylB* genes from *E. coli* MG1655, *P. polymyxa* SC2, *S. coelicolor,* and *X. campestris* 8004 (designations and sources are listed in Table 2) were cloned into the IPTG-inducible expression vector pXMJ19. The resulting plasmids pX-ecoAB, pX-ppmAB, pX-scoAB, and pX-xcbAB, were individually introduced into *C. glutamicum* ATCC 13032 along with pXMJ19 to generate the corresponding recombinant strains I-eco, I-ppm, I-sco, I-xcb, and I-pXMJ19, respectively. The growth and xylose consumption of the strains are shown in Fig. 2a.

**Table 2:** Codon usage analysis of *xylA* and *xylB* orthologs from different sources

Gene	Source	Gene symbol	Protein size (kDa)	Codon usage	
				Rarely used codons	Very rarely used codons
*xylA*	*E. coli*	b3565	47.5	7	1
	*P. polymyxa*	PPSC2_c4572	52.2	7	2
	*S. coelicolor*	SCO1169	41.8	23	0
	*X. campestris*	XC_2477	49.2	5	0
*xylB*	*E. coli*	b3564	52.3	29	2
	*P. polymyxa*	PPSC2_c4571	54.8	17	2
	*S. coelicolor*	SCO1170	51.9	26	0
	*X. campestris*	XC_2478	54.8	5	0

‘Very rarely used codons’ and ‘Rarely used codons’ indicate codons represented at less than 10% or 20% in the codon usage table of *C. glutamicum* (http://www.kazusa.or.jp/codon/cgi-bin/showcodon.cgi?species=196627&aa=1&style=N)

Owing to the lack of xylose isomerase, the control strain I-pXMJ19 could not grow on xylose, as expected. By contrast, all the recombinant strains with the XI pathway were able to grow on xylose as sole carbon source. Among them, strain I-xcb exhibited a markedly faster maximum specific growth rate (0.224 ± 0.003 h^−1^) and average xylose consumption rate (0.44 g L^−1^ h^−1^, calculated at 30 h) than the other three strains. This observation was in agreement with a previous report, in which the overexpression of *xylA* from *X. campestris* and the endogenous *xylB* endowed *C. glutamicum* with a fast growth rate (0.199 ± 0.009 h^−1^) [23]. SDS-PAGE analysis of intracellular proteins showed that strain I-xcb had the highest protein expression of xylose isomerase and xylulokinase (Fig. 2b). Furthermore, codon usage analysis revealed that *xylAB* from *X. campestris* has the most similar codon usage to that of *C. glutamicum* (Table 2), which might be the main reason for the observed high protein expression level.

In view of the different profiles of aerobic and anaerobic metabolism, the xylose utilization of strains I-eco, I-ppm, I-sco, and I-xcb was also evaluated under anaerobic conditions (Fig. 2c). Similar with the results obtained under aerobic conditions, strain I-xcb exhibited a faster xylose consumption rate (1.80 g L^−1^ h^−1^, calculated at 12 h) than the other three strains (1.62 g L^−1^ h^−1^ for strain I-eco, 0.57 g L^−1^ h^−1^ for strain I-ppm and 0.65 g L^−1^ h^−1^ for strain I-sco).

It has been proved that overexpression of pyruvate carboxylase (*pyc*), citrate synthase (*gltA*), and succinate exporter (*sucE*) could efficiently improve succinate production in *C. glutamicum* [15], and therefore, plasmid pEpycgltAsucE (overexpressing *pyc*, *gltA,* and *sucE* genes) was introduced into SAZ3 (Δ*ldhA*Δ*pta*Δ*pqo*Δ*cat*P_sod_-*ppc*P_sod_-*pyc*) along with the plasmid pX-xcbAB. The resulting strain CGS1 was further tested for succinate production.

### Effect of the carbon source used during the two-stage fermentation on succinate production

The carbon source influences gene expression of metabolic networks and, consequently, affects succinate production during the fermentation. It has been proven that the succinate yield from xylose is significantly higher than from glucose (25% versus 14%) in *C. glutamicum* under anaerobic conditions [19], suggesting that xylose might be a better substrate for succinate production. However, it has been demonstrated that the supply of ATP from xylose metabolism is insufficient during anaerobic succinate production in *E. coli* [7], and glucose was recommended as a co-substrate to alleviate the observed ATP shortage [36]. Therefore, the effect of carbon source used during the anaerobic phase on succinate production was evaluated in CGS1.

First, we prepared seed cultures using glucose as carbon source (in CGXIIA-G seed medium) in the first stage, after which the seed cultures were collected for succinate production during the anaerobic stage in which glucose or xylose was used as substrate. As shown in Table 3, the deletion of *ldh* completely abolished lactate accumulation in CGS1. Notably, as the substrate used in the second stage, xylose was clearly superior to glucose in both succinate titer (27.4 versus 24.6 g L^−1^) and yield (0.90 versus 0.81 g L^−1^). Compared with succinate production from glucose, we also observed a dramatic decrease of pyruvate accumulation (from 2.19 to 0.03 g L^−1^) when xylose was fermented. This is most likely due to the fact that the phosphotransferase system (PTS) was not used for xylose transport, which otherwise would form pyruvate. Since the major acetate synthetic pathways were abolished in CGS1, with the exception of pyruvate complex dehydrogenase E1 component (encoded by *aceE*) (Fig. 1), the acetate accumulation was limited to a relatively low level. There was no obvious difference in acetate production between glucose and xylose fermentation (0.45 versus 0.46 g L^−1^). Liu et al. [7] discovered that *E. coli* needs to alleviate an ATP shortage to enable cell growth under anaerobic conditions by acetate synthesis during xylose fermentation. However, the cell growth of *C. glutamicum* was arrested under oxygen deprivation [37], and the demand for ATP was, therefore, not as strong as that in *E. coli*, which might be the reason for the similar acetate accumulation. α-Ketoglutarate, synthesized in the tricarboxylic acid cycle (TCA cycle), has been reported as a major by-product during succinate production in *C. glutamicum* [5, 14, 15]. It is worth mentioning that malate dehydrogenase (encoded by *mdh*) is almost equally active with NADH and NADPH as coenzyme, judging by their respective *K*_cat_ values [38]. This means that NADPH produced from the accumulation of α-ketoglutarate could also serve as an efficient source of reducing power for the production of succinate. Interestingly, α-ketoglutarate accumulation during xylose fermentation was also 24.7% lower than during glucose fermentation (1.34 versus 1.78 g L^−1^). The reason for this remains unknown.

**Table 3:** Succinate production from xylose by CGS1 under anaerobic conditions

Strain	Substrate in seed culture	Substrate in anaerobic fermentation	Sugar consumption rate (g L^−1^ h^−1^)^a^	Succinate yield (g g^−1^ sugar)	Consumed sugars (g L^−1^)	Titer (g L^−1^)					
						Succinate	α-Ketoglutarate	Pyruvate	Lactate	Fumarate	Acetate
CGS1	Glucose	Glucose	2.75 ± 0.03	0.81 ± 0.01	30.5 ± 0.4	24.6 ± 0.1	1.78 ± 0.01	2.19 ± 0.03	ND	ND	0.46 ± 0.01
		Xylose	1.39 ± 0.02	0.90 ± 0.01	30.5 ± 0.3	27.4 ± 0.3	1.34 ± 0.03	0.24 ± 0.08	ND	ND	0.45 ± 0.01
	Xylose	Xylose	1.95 ± 0.02	0.93 ± 0.02	30.4 ± 0.3	28.2 ± 0.3	1.32 ± 0.01	0.29 ± 0.05	ND	ND	1.14 ± 0.01
		Glucose	2.33 ± 0.04	0.93 ± 0.01	30.1 ± 0.2	28.1 ± 0.1	2.09 ± 0.01	ND	ND	ND	1.69 ± 022

Values are given as the averages and standard deviations of three independent cultures

Succinate production was carried out with the same initial cell density (OD_600_ = 30). 30 g L^−1^ 4MgCO_3_·Mg(OH)_2_·5H_2_O, 200 mM sodium bicarbonate, and 1 mM IPTG were added to the medium

*ND* not detected

^a^Sugar consumption rates were calculated at 8 h before the sugars were completely depleted

It is notable that the xylose consumption rate of strain CGS1 was 50.5% lower than its glucose consumption rate during the second stage (1.39 versus 2.75 g L^−1^ h^−1^, Table 3) when the seed cultures were prepared using glucose as substrate. Thus, to improve the xylose consumption rate under anaerobic conditions, xylose was used as substrate for the seed culture in CGXIIA-X medium, and its effect on succinate production and xylose utilization was evaluated. Perhaps, unsurprisingly, the xylose consumption rate of CGS1 in the second stage (1.95 g L^−1^ h^−1^) was improved by 40.3% when xylose was used for the seed culture, and a slight improvement of succinate yield was also observed (0.93 versus 0.90 g g^−1^). There was no obvious difference in the accumulation of α-ketoglutarate and pyruvate in fermentations conducted with seed cultures from CGXIIA-G and CGXIIA-X medium. However, CGS1 collected from CGXIIA-X medium produced a 1.5-fold higher acetate concentration (1.14 g L^−1^) than when collected from CGXIIA-G medium (0.45 g L^−1^), which might be due to the aforementioned ATP shortage observed in aerobic seed cultures with xylose as carbon source.

To fully evaluate the effects of the carbon source, seed cultures from CGXIIA-X medium were also used for succinate production in the anaerobic stage with glucose as substrate. Compared to succinate production by seed cultures from CGXIIA-G medium, the glucose consumption rate of CGS1 collected from CGXIIA-X medium was decreased by 15.3% (2.33 versus 2.75 g L^−1^ h^−1^, Table 3). However, both the succinate titer (28.1 versus 24.6 g L^−1^) and yield (0.93 versus 0.81 g g^−1^) were significantly improved.

**Fig. 3:**
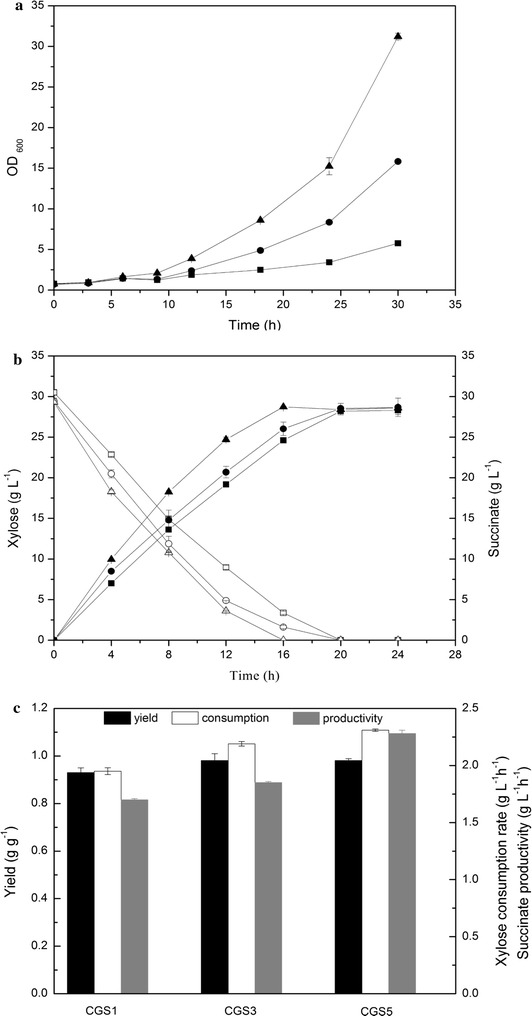
Batch fermentation of *C. glutamicum* CGS1 (squares), CGS3 (circles) and CGS5 (upward triangles). **a** Profiles of cell growth (filled symbols) and xylose consumption (open symbols) in CGXIIA medium with 30 g L^−1^ xylose. **b** Profiles of succinate production (filled symbols) and xylose consumption (open symbols) under anaerobic conditions with an initial OD_600_ of 30. 30 g L^−1^ 4MgCO_3_·Mg(OH)_2_·5H_2_O, 200 mM sodium bicarbonate, and 1 mM IPTG were added into the CGXIIB medium. **c** Profiles of succinate yield (black bars), xylose consumption rate (white bars, calculated at 8 h), and succinate productivity (grey bars, calculated at 8 h) under anaerobic conditions

As these results demonstrate, an effect of carbon source was inevitable during succinate production under oxygen deprivation, and xylose was preferable to glucose both during the aerobic and the anaerobic fermentation phase. As shown in Fig. 3b, c, CGS1 was able to produce succinate with a good yield (0.93 g g^−1^ d-xylose, 83% of the theoretical yield), while its productivity (1.70 g L^−1^ h^−1^ at 8 h) was still not satisfactory. Hence, further genetic manipulations were carried out to improve the xylose utilization rate and succinate productivity.

### Improvement of xylose utilization and succinate production by the expression of *tkt*, *tal,* and a xylose transporter

It was reported that overexpression of *tkt* (encoding transketolase) and *tal* (encoding transaldolase) increased the flux from the non-oxidative PPP into the glycolytic pathway, and improved the rate of xylose assimilation [39, 40]. Recently, Jo et al. [28] demonstrated that overexpression of *tal* in *C. glutamicum* could efficiently improve xylose utilization and succinate production during an aerobic fermentation process. Hence, to further facilitate xylose utilization, the strong constitutive promoter *sod* was inserted in front of *tkt* and *tal* in strain SAZ3, yielding strain CGS2. The plasmid pX-xcbAB was introduced into CGS2 along with pEpycgltAsucE to generate CGS3. Under anaerobic conditions, the transcriptional levels of *tkt* and *tal* in CGS3 were increased 13.08- and 5.77-fold, respectively, compared with strain CGS1.

As shown in Fig. 3a, CGS3 had an obvious growth advantage over CGS1 during aerobic xylose fermentation, which was consistent with a previous report [28]. During the anaerobic fermentation, overexpression of *tkt* and *tal* also improved the xylose consumption rate by 12.3% (2.19 versus 1.95 g L^−1^ h^−1^ at 8 h). Consequently, the succinate productivity increased from 1.70 to 1.85 g L^−1^ h^−1^. In addition, the yield of succinate was also slightly improved from 0.93 to 0.98 g g^−1^, reaching 88% of the theoretical yield.

Engineering of sugar transport was a driving force for efficient bioconversion of sugar mixtures derived from lignocellulose [41]. It is notable that the consumption rates of pentose sugars in *C. glutamicum* decreased appreciably at low sugar substrate concentrations [19, 42], which would encumber the whole fermentation process and cause a low production rate of succinate. To address this, the pentose transporter gene *araE* from *Bacillus subtilis* [43] was integrated into the chromosome of strain CGS2 at the *ldh* locus, yielding the strain CGS4. Subsequently, the plasmid pX-xcbAB was introduced into CGS4 along with pEpycgltAsucE to generate CGS5.

As expected, CGS5 showed a further improvement in growth under aerobic conditions (Fig. 3a). During the anaerobic fermentation, the yield of succinate remained at 0.98 g g^−1^, and the xylose consumption rate was slightly improved from 2.19 to 2.31 g L^−1^ h^−1^. Notably, the succinate production rate was significantly improved from 1.85 to 2.28 g L^−1^ h^−1^. The improvement of the succinate production rate was relatively higher than that of the xylose consumption rate. This was consistent with a previous report in which the recombinant strain CRX-araE showed 4.2-fold higher lactate productivity with a 2.7-fold higher xylose consumption rate after introducing *araE* from *C. glutamicum* ATCC 31831 [24].

### Succinate production from a mixture of glucose and xylose

To evaluate the feasibility of anaerobic succinate production from different kinds of lignocellulosic biomass, strain CGS5 was cultured in two-stage fermentations, and CGXIIB media with different ratios of glucose to xylose were used in the second stage. As shown in Table 4, there were no obvious differences in succinate titer and yield for the four sugar ratios. The yield of succinate reached very high levels, from 0.97 to 1.00 g g^−1^. The production of the major by-product α-ketoglutarate varied with the sugar ratios, while the titer of acetate remained at 0.5 g L^−1^. The robustness of succinate production from the mixtures with different sugar ratios demonstrated that the engineered strain has great potential for the utilization of lignocellulosic hydrolysates of all kinds of biomass.

**Table 4:** Succinate production from sugar mixtures with different ratios of glucose to xylose by CGS5 under anaerobic conditions

Strain	Succinate yield	Consumed sugars (g L^−1^)		Titer (g L^−1^)					
	(g/g total sugars)	Glucose	Xylose	Succinate	α-Ketoglutarate	Pyruvate	Lactate	Fumarate	Acetate
CGS5	0.98 ± 0.01	0	30.4 ± 0.3	29.7 ± 0.1	1.41 ± 0.01	0.31 ± 0.01	ND	ND	0.66 ± 0.01
	0.98 ± 0.03	10.6 ± 0.1	21.1 ± 0.2	30.9 ± 0.6	1.04 ± 0.36	0.24 ± 0.09	ND	ND	0.65 ± 0.02
	0.97 ± 0.05	15.7 ± 0.3	15.0 ± 0.4	29.7 ± 0.9	0.70 ± 0.09	0.19 ± 0.04	ND	ND	0.59 ± 0.02
	1.00 ± 0.03	21.6 ± 0.1	10.1 ± 0.1	31.8 ± 0.3	1.24 ± 0.06	0.27 ± 0.01	ND	ND	0.63 ± 0.01
	1.00 ± 0.01	31.5 ± 0.1	0	31.4 ± 0.1	0.80 ± 0.02	0.38 ± 0.01	ND	ND	0.52 ± 0.01

Values are given as the averages and standard deviations of three independent cultures

Succinate production was carried out with the same initial cell density (OD_600_ = 30). 30 g L^−1^ 4MgCO_3_·Mg(OH)_2_·5H_2_O, 200 mM sodium bicarbonate, and 1 mM IPTG were added to the medium

*ND* not detected

**Fig. 4:**
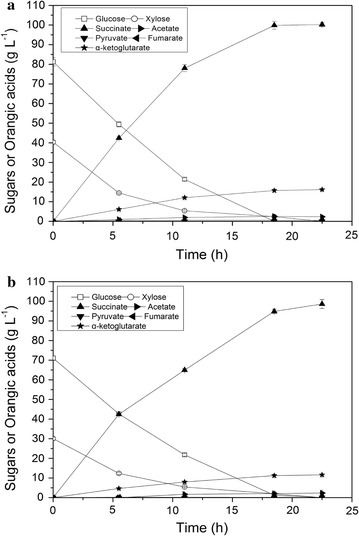
Succinate production by CGS5 at high cell density (OD_600_ = 150) under anaerobic conditions. 100 g L^−1^ 4MgCO_3_·Mg(OH)_2_·5H_2_O, 300 mM sodium bicarbonate, and 2 mM IPTG were added into the CGXIIB medium. The consumption of glucose (open squares), xylose (open circles), and concentration of succinate (filled upward triangles), ketoglutarate (filled stars), pyruvate (filled downward triangles), fumarate (filled leftward triangles), and acetate (filled rightward triangles) are shown. **a** Profiles of the production of organic acids and consumption of sugars from mixtures of glucose and xylose. **b** Profiles of the production of organic acids and consumption of sugars from lignocellulosic hydrolysate

A high sugar concentration can lower the operating costs of industrial processes by reducing the time required for feeding and the risk of contamination. To test the strain’s ability to produce succinate at high sugar concentrations, we used CGXIIB medium supplemented with 81.3 g L^−1^ glucose and 40.3 g L^−1^ xylose to culture CGS5 in the second stage. The ratio of glucose to xylose (2:1) was consistent with the glucose/xylose ratio of the most common lignocellulosic hydrolysate. As shown in Fig. 4a, CGS5 simultaneously and completely consumed glucose and xylose without significant carbon catabolite repression (CCR) under anaerobic conditions. 100.2 g L^−1^ succinate was accumulated within 23 h, with a productivity of 4.35 g L^−1^ h^−1^. Okino et al. [13] observed that the succinate yield (about 1.45 mol/mol glucose) of *C. glutamicum* ΔldhA-pCRA717 was not affected by the glucose concentration (from 20 to about 280 mM). Notably, when the glucose concentration was subsequently increased to 420 mM (75.6 g L^−1^), the succinate yield was decreased by about 15.2% to 1.23 mol/mol. Our results showed that the yield of succinate from a high-concentration sugar mixture (121.6 g L^−1^ total sugars) was 18.0% lower than that from a low-concentration mixture (31.7 g L^−1^ total sugars) with the same glucose/xylose ratio (0.82 versus 1.00 g/g total sugars), which was consistent with the aforementioned report [13]. However, the loss of carbon yield can be attributed to the soaring accumulation of α-ketoglutarate, since its titer increased from 1.24 to 16.2 g L^−1^ and its yield increased from 0.04 to 0.13 g g^−1^ total sugars, which means that the activity of the oxidative branch of the TCA cycle was also drastically increased. Because of the similar fermentation conditions, the residual oxygen in serum bottles could not answer for the variation of TCA activity. Even under strict anaerobic condition, a small but statistically significant flux through the oxidative part of the TCA cycle in wild-type *C. glutamicum* has been observed by bubbling CO_2_ instead of argon [44], which means that the activity of oxidative TCA arm does exist and can be activated under anaerobic conditions. This does make sense in view of the overall redox balance [44], but the reason for the variation of TCA activity is still unknown. In addition, acetate increased to 2.3 g L^−1^, but the acetate yield decreased, which suggested that the ATP demand was actually reduced at the higher sugar concentration. The concentration of fumarate reached 0.55 g L^−1^, while the concentrations of other organic acids were below 0.1 g L^−1^, which was negligible. However, the succinate yield of 0.82 g g^−1^ was still acceptable and was obtained alongside a titer of 100.2 g L^−1^.

### Efficient succinate production from lignocellulosic hydrolysate

Under oxygen deprivation, *C. glutamicum* exhibits high tolerance to general lignocellulose-derived fermentation inhibitors [45]. However, inhibitors such as furfural, 5-hydroxymethylfurfural and vanillic had an inhibitory effect on glucose consumption and succinate accumulation under anaerobic conditions, and their mixtures collocated as the actual diluted acid hydrolysate of corn cobs showed a more toxic effect on succinate production [46]. To avoid this problem, we used corn stalk hydrolysate with much less inhibitors, which was prepared using the most widely used commercial cellulolytic enzyme reagent Cellic^®^ CTec2 [47]. In the second stage, CGXIIB medium with diluted hydrolysate (71.0 g L^−1^ glucose and 30.1 g L^−1^ xylose) was used for succinate production.

There were no significant differences in the sugar utilization pattern between the fermentations with mixed sugars (Fig. 4a) and the hydrolysate (Fig. 4b), but the consumption rate of total sugars decreased by 16.8% (4.40 versus 5.29 g L^−1^ h^−1^). However, to our surprise, 98.6 g L^−1^ succinate was accumulated with a yield of 0.98 g/g total sugars, which was 16.3% higher than the yield of 0.82 g/g total sugars generated from the mixed sugars. The volumetric productivity of succinate from lignocellulosic hydrolysate was, therefore, comparable to that from sugar mixture (4.29 versus 4.35 g L^−1^ h^−1^). Likewise, 11.6 g L^−1^ of α-ketoglutarate and 2.4 g L^−1^ of acetate were accumulated as major by-products. Although the titer of α-ketoglutarate was reduced, the yield of α-ketoglutarate was not significantly decreased (0.11 versus 0.13 g/g total sugars). The concentration of fumarate was 0.15 g L^−1^ and that of other organic acids was less than 0.1 g L^−1^. Other sugars (not completely hydrolyzed disaccharides and other monosaccharides) generated during enzymatic hydrolysis might be one reason for the increased succinate yield, although they were not identified. The main reason could be the presence of citric acid/sodium citrate in the medium (the citric acid concentration in CGXIIB was about 22.1 g L^−1^), which was used as the enzyme buffer during the preparation of corn stalk hydrolysate. After cultivation for 22.5 h, 12.4 g L^−1^ of citric acid (64.5 mM) were consumed for succinate production, which simulated a higher succinate yield from total sugar. As shown in Fig. 1, the carbon flux supposed to flow to the oxidative branch of the TCA cycle could be saved by consuming citrate to produce α-ketoglutarate and NADPH. The succinate yield based on the total sugars was, therefore, increased; however, to be more accurate, the succinate yield was recalculated as 0.87 g/g total substrates, based on the consumed glucose, xylose, and citrate.

As far as we know, 98.6 g L^−1^ succinate is the highest ever reported titer obtained from non-food lignocellulosic hydrolysate (Table 5), and the yield and productivity were also good enough to merit further industrial scale-up.

**Table 5:** Production of succinate from non-food lignocellulosic biomass

Strain	Substrate (treatment)	Conditions	Titer (g/L)	Yield (g/g)	Productivity (g L^−1^ h^−1^)	References
*Actinobacillus succinogenes*						
CGMCC1593	Corn straw (enzymatic hydrolysis)	One stage, anaerobic fed-batch fermentation, complex medium	53.2	0.83	1.21	[48]
CGMCC1593	Corn stover (enzymatic hydrolysis)	One stage, anaerobic batch fermentation, complex medium	47.4	0.72^a^	0.99	[49]
NJ113	Corn fiber (dilute acid hydrolysis)	One stage, anaerobic batch fermentation, complex medium	35.4	0.73	0.98	[50]
NJ113	Sugarcane bagasse (dilute acid hydrolysis)	One stage, anaerobic batch fermentation, complex medium	23.7	0.79	0.99	[51]
NJ113	Corn fiber hydrolysate (dilute acid hydrolysis)	One stage, anaerobic batch fermentation, complex medium	47.6	0.68	0.63	[9]
NJ113	Corn stover (enzymatic hydrolysis)	One stage, anaerobic batch fermentation, complex medium	56.4	0.73	1.08	[52]
130Z	Corn stover (dilute acid hydrolysis, deacetylation)	One stage, anaerobic continuous fermentation, complex medium	39.6	0.78	1.77	[53]
130Z	Corn stover (dilute acid hydrolysis, deacetylation)	One stage, anaerobic batch fermentation, complex medium	42.8	0.74	1.27	[54]
130Z	Pinewood (enzymatic hydrolysis)	One stage, anaerobic batch fermentation, complex medium	20.7	0.65	0.90	[55]
CIP 106512	Sugarcane bagasse hemicellulose (dilute acid hydrolysis)	One stage, anaerobic batch fermentation, complex medium	22.5	0.43	1.01	[56]
*Escherichia coli*						
BA204	Corn stalk (dilute acid hydrolysis)	Two stages, aerobic culture of biomass and anaerobic batch fermentation, complex medium	11.1	1.02	0.70	[7]
AFP 184	Spruce softwood (dilute acid hydrolysis)	Two stages, aerobic culture of biomass and anaerobic batch fermentation, complex medium	42.2	0.72	0.78	[57]
SD121	Corn stalk (enzymatic hydrolysis)	Two stages, aerobic culture of biomass and anaerobic batch fermentation, complex medium	57.8	0.87	0.96	[8]
BA305	Sugarcane bagasse (enzymatic hydrolysis)	Two-stages, aerobic culture of biomass and anaerobic batch fermentation, Cell-recycling repeated fermentation, minimal medium	83^b^	0.87	2.31	[10]
BA305	Sugarcane bagasse (not mentioned)	One stage, anaerobic fed-batch fermentation, complex medium	39.3	0.97	0.33	[58]
DC1515	Corn stalk (enzymatic hydrolysis)	One stage, anaerobic batch fermentation, complex medium	38.6	0.39	0.80	[59]
*Anaerobiospirillum succiniciproducens*	Oak wood (enzymatic hydrolysis)	One stage, anaerobic batch fermentation, complex medium	24	0.88	0.75	[60]
*Mannheimia succiniciproducens*	Oak wood (enzymatic hydrolysis)	One stage, anaerobic batch fermentation, complex medium	11.7	0.56	1.17	[61]
*Basfia succiniciproducens*	Corn stover (dilute acid hydrolysis)	One stage, anaerobic batch fermentation, complex medium	30	0.69	0.43	[62]
*Corynebacterium crenatum*	Wheat bran (dilute acid hydrolysis)	Two stages, aerobic culture of biomass and anaerobic batch fermentation, Cell-recycling repeated fermentation, minimal medium	43.6	0.59	4.36	[63]
*Corynebacterium glutamicum*						
NC-2	Corn Cob (dilute acid hydrolysis)	Two-stage, aerobic culture of biomass and anaerobic batch fermentation, minimal medium	40.8	0.69	0.85	[18]
CGS5	Corn stalk (enzymatic hydrolysis)	Two-stage, aerobic culture of biomass and anaerobic batch fermentation, minimal medium	98.6	0.87^c^	4.29	This study

^a^This yield was defined as the amount of succinic acid produced per substrate consumed

^b^The titer of 83 g L^−1^ was calculated by summing up the titers of three repetitive fermentations

^c^This yield was calculated based on the consumed glucose, xylose, and citrate

## Conclusions

In summary, an efficient two-stage fermentation process for succinate production from lignocellulosic hydrolysate, with a high titer, yield, and productivity, was achieved by metabolically engineering *C. glutamicum*. A titer of 98.6 g L^−1^ succinate was obtained with a yield of 0.87 g/g total substrates and a succinate productivity of 4.29 g L^−1^ h^−1^. The excellent utilization of lignocellulosic hydrolysate and efficient succinate production under anaerobic conditions highlight *C. glutamicum*, which is a biosafety level 1 microorganism, as a promising chassis for the lignocellulosic biorefinery.

## Additional file

**Additional file 1: Table S1.** Primers used in this study.
