# Supplementary material for: Metabolic engineering of Corynebacterium glutamicum for efficient production of succinate from lignocellulosic hydrolysate
Source: Biotechnol Biofuels. 2018 Apr 4;11:95. doi: 10.1186/s13068-018-1094-z (PMC5883316; doi:10.1186/s13068-018-1094-z)
Supplement: Supplementary file 1 — Additional file 1: Table S1. Primers used in this study. [file 13068_2018_1094_MOESM1_ESM.docx]

**Metabolic engineering of** ***Corynebacterium glutamicum* for efficient production of succinate from lignocellulosic hydrolysate**

Yufeng Mao^a,^*, Guiying Li^a,^*, Zhishuai Chang^a,^*, Ran Tao^a^, Zhenzhen Cui^a^, Zhiwen Wang^a,▼^, Ya-jie Tang^b^, Tao Chen^a,▼^, Xueming Zhao^a^

^a^ Key Laboratory of Systems Bioengineering (Ministry of Education); SynBio Research Platform, Collaborative Innovation Center of Chemical Science and Engineering (Tianjin); School of Chemical Engineering and Technology, Tianjin University, Tianjin, 300072, China.

^b^ Key Laboratory of Fermentation Engineering, Ministry of Education, Hubei University of Technology, Wuhan 430068, China

* These authors contribute equally to this work.

▼Corresponding author: Zhiwen Wang

E-mail: zww@tju.edu.cn

Tel: +86 22 27406770; Fax: +86 22 27406770.

▼Corresponding author: Tao Chen

E-mail: [chentao@tju.edu.cn](mailto:chentao@tju.edu.cn)

Tel: +86 22 27406770; Fax: +86 22 27406770.

**Table S1** Primers used in this study

| Primers | Sequence | Purpose |
| --- | --- | --- |
| ecoxylAB-1 | TAGCAAGCTTAAAGGAGGACAACCATGCAAGCCTATTTTGACCAGCTC | To construct pX-ecoAB |
| ecoxylAB-2 | GAGCTCTAGACAGGGGATAACGTTTACGCCATTA | To construct pX-ecoAB |
| ppmxylAB-1 | TCGACTCTAGAAAAGGAGGACAACCATGGCTTATTTCGAACAAGTATCC | To construct pX-ppmAB |
| ppmxylAB-2 | AGCTCGGTACCCGGGGATCCACCCTTATTATGGCTTTTATT | To construct pX-ppmAB |
| scoxylA-1 | TGGTCTGCAGAAAGGAGGACAACCCGGAATGAACTACCAGCCCACT | To construct pX-scoAB |
| scoxylA-2 | CTTTTCTAGAACGGGGCCGGACATTCTCAC | To construct pX-scoAB |
| scoxylB-1 | TGAGAATGTCCGGCCCCGTTCTAGAAAAGGAGGACAACCAAGCCCGATGTCAGCAGCCG | To construct pX-scoAB |
| scoxylB-2 | TCTTGGTACCCGACGGGAGATGCCTGAGAT | To construct pX-scoAB |
| xcbxylA-1 | TGTAAGCTTAAAGGAGGACAACCATGAGCAACACCGTTTTCATCGG | To construct pX-xcbAB |
| xcbxylA-2 | CGCTCATGGTTGTCCTCCTTTGGTACCACATCAACGCGTCAGGTACT | To construct pX-xcbAB |
| xcbxylB-1 | ACCAAAGGAGGACAACCATGAGCGTGTACGTAGGACT | To construct pX-xcbAB |
| xcbxylB-2 | CTGTCTAGACCTTGGTACTGGGGGAGTGATGAAT | To construct pX-xcbAB |
| P_sod_-1 | TAGCTGCCAATTATTCCGGG | To amplify *sod* promoter |
| P_sod_-2 | GGGTAAAAAATCCTTTCGTAG | To amplify *sod* promoter |
| PSL-1 | ACGAATTCGAGCTCGGTACCCGGGGATCCTCTAGGAAACCCCAGATGTGATCCTC | To construct pD-PSL |
| PSL-2 | GGGTCACAAGCCCGGAATAATTGGCAGCTACCTTAAAAGAATTGTTCTTGAACTG | To construct pD-PSL |
| PSL-3 | TGCGGAAACCTACGAAAGGATTTTTTACCCATGTCTCACATTGATGATCTTGCAC | To construct pD-PSL |
| PSL-4 | GCCAAGCTTGCATGCCTGCAGGTCGACTCTAGCTGCTTGATGCCCTCGATGAAC | To construct pD-PSL |
| PSK-1 | ACGAATTCGAGCTCGGTACCCGGGGATCCTCTAGCAAAACTCGCGCGGAACCAGACCTT | To construct pD-PSK |
| PSK-2 | GGGTCACAAGCCCGGAATAATTGGCAGCTATCCTTCCTGGGTTAAACCGGGA | To construct pD-PSK |
| PSK-3 | TGCGGAAACCTACGAAAGGATTTTTTACCCATGACCACCTTGACGCTGT | To construct pD-PSK |
| PSK-4 | GCCAAGCTTGCATGCCTGCAGGTCGACTCTAGCAGAAGCAATGACGTAGATGTG | To construct pD-PSK |
| P_tuf_-1 | AATCGAATTCTGGCCGTTACCCTGCGAATG | To amplify *tuf* promoter |
| P_tuf_-2 | ATCAGTCGACGAGCTCCGATAGGATCCAGGTATCTAGATGTATGTCCTCCTGGACTTCG | To amplify *tuf* promoter |
| araE-1 | ACGCTTCTAGAAAAGGAGGACAACCATGAAGAATACTCCAACTC | To construct pD-P_tuf_araE |
| araE-2 | GACGTCGACTATAAACAGCCCTTCCCGTAG | To construct pD-P_tuf_araE |
| ldh-F1 | ATGCGAATTCCAAGGTGCCGACACTAATGC | To construct pD-P_tuf_araE |
| ldh-F2 | ATAGGTCGACGGCCCCTGCAGGGCCCGATTATGTCCGAAAATGT | To construct pD-P_tuf_araE |
| ldh-B1 | AGTAGTCGACTCAATGCATATGATCTTTGGCGCCTAGTTGGC | To construct pD-P_tuf_araE |
| ldh-B2 | TATCAAGCTTGCTTCCAGACGGTTTCATCG | To construct pD-P_tuf_araE |
| 16srRNA-1 | GGAGAAGAAGCACCGGCTAA | RT-*q*PCR |
| 16srRNA-2 | ACGCTCGCACCCTACGTATT | RT-*q*PCR |
| RT-tal-1 | TGTGGCTTCCTTCTTCGTC | RT-*q*PCR |
| RT-tal-2 | CAGAGCCAAAGCCTCATCG | RT-*q*PCR |
| RT-tkt-1 | ACTGTACGCAATTACCCCTC | RT-*q*PCR |
| RT-tkt-2 | CCACAGTTTTCTACAGCGTCT | RT-*q*PCR |
